# Multivariate Assessment of Thyroid, Lipid, and Inflammatory Profiles by HBV Status and Viral Load: Age- and Sex-Specific Findings

**DOI:** 10.3390/v17091208

**Published:** 2025-09-03

**Authors:** Hyeokjun Yun, Jong Wan Kim, Jae Kyung Kim

**Affiliations:** 1 Department of Medical Laser, Dankook University School of Medicine, Cheonan-si 31116, Republic of Korea; 10621yhj@naver.com; 2 Department of Laboratory Medicine, Dankook University School of Medicine, Cheonan-si 31116, Republic of Korea; wan1818@paran.com; 3 Department of Biomedical Laboratory Science, Dankook University College of Health Sciences, Cheonan-si 31116, Republic of Korea

**Keywords:** C-reactive protein (CRP), cholesterol HDL, cholesterol LDL, hepatitis B virus (HBV), thyrotropin (TSH)

## Abstract

Chronic hepatitis B virus (HBV) infection may influence extrahepatic systems, including endocrine and lipid regulation. In this cross-sectional study, 186 adults were stratified by HBV DNA status and viral load to examine thyroid function, systemic inflammation, and lipid metabolism, with further analyses by age and sex. Thyroid-stimulating hormone (TSH, a pituitary regulator of thyroid function) levels were significantly lower in HBsAg-positive individuals compared with controls; however, this association was attenuated after stratification by viral load, indicating that the relationship is not unequivocally independent of HBV DNA levels, as free thyroxine (FT4, the circulating thyroid hormone reflecting gland activity) levels remained stable. Lipid profiles displayed demographic-specific patterns: males with high viral load exhibited lower HDL cholesterol, whereas younger HBV-positive individuals showed higher LDL cholesterol. CRP levels were unaffected by HBV status or viral load, aligning with the absence of systemic inflammation in early or inactive disease stages. Age was a major determinant across biomarkers, with complex interactions involving sex and viral load. These findings indicate subtle but clinically relevant extrahepatic effects of HBV infection and underscore the need for personalized monitoring and longitudinal studies to clarify metabolic and cardiovascular implications. These subgroup trends should be interpreted with caution given the absence of BMI, liver enzyme, fibrosis, medication, and comorbidity data in this retrospective cohort.

## 1. Introduction

Chronic hepatitis B virus (HBV) infection remains a significant global health burden, with an estimated 296 million people affected worldwide and nearly 820,000 deaths annually, primarily due to cirrhosis and hepatocellular carcinoma [1]. While the hepatic manifestations of chronic HBV infection are well-established, recent research has highlighted its potential extrahepatic effects, including disturbances in endocrine, inflammatory, and metabolic pathways [2,3,4]. Given the liver’s central role in hormone metabolism, lipid regulation, and systemic inflammation, chronic infection with HBV may disrupt these physiological processes even in the absence of advanced liver disease [5].

HBV is a small, enveloped, partially double-stranded DNA virus belonging to the family *Hepadnaviridae*. It primarily infects hepatocytes and establishes persistence through covalently closed circular DNA (cccDNA) in the nucleus [6]. HBV is transmitted via perinatal exposure, unprotected sexual contact, and parenteral routes such as blood transfusion or use of contaminated needles [1,6]. These virological characteristics underlie its chronicity and widespread distribution, contributing to its major public health impact.

The infectious HBV virion, or Dane particle (~42 nm), consists of a lipid envelope containing surface proteins (small, middle, and large S; collectively HBsAg) that mediate host entry, surrounding a nucleocapsid composed of hepatitis B core antigen (HBcAg). The capsid encloses the partially double-stranded DNA genome and viral polymerase, essential for replication. In addition, the precore protein is secreted as HBeAg, a marker of replication and immune tolerance, but not part of the virion structure [1,6,7].

Previous studies have suggested that HBV infection may alter thyroid function, particularly by modulating the hypothalamic–pituitary–thyroid (HPT) axis. Reduced TSH levels have been observed in HBV-infected individuals, sometimes in the absence of significant changes in FT4, raising questions about subclinical central suppression or early endocrine adaptation to chronic antigen exposure [5,8]. In addition, the inflammatory profile in HBV infection, commonly assessed by serum C-reactive protein (CRP), appears to vary depending on the phase of infection, with higher CRP levels reported during immune-active states and in association with liver damage [9,10,11]. However, findings remain inconsistent, possibly due to differences in host immune status, viral load, or demographic factors.

Lipid metabolism is another domain potentially affected by chronic HBV infection. Alterations in lipid parameters—including high-density lipoprotein (HDL), low-density lipoprotein (LDL), and triglycerides—have been reported, although the direction and magnitude of changes vary across studies [12,13,14]. HBV may interfere with hepatic lipid processing through direct effects on hepatocytes or indirectly via chronic inflammation and immune activation. These metabolic changes may carry important clinical implications, particularly in light of increasing awareness of cardiovascular risk and metabolic syndrome in patients with chronic liver disease [15]. These biomarkers were selected due to their reported susceptibility to modulation by chronic viral infection, hepatic metabolic regulation, and systemic inflammation [5,12,15].

Despite these observations, integrated analyses of endocrine, inflammatory, and lipid profiles in relation to HBV infection remain limited [16]. Few studies have examined the combined effects of HBV DNA status, viral load, age, and sex on these systemic parameters [17]. A better understanding of how these factors interact could offer insights into HBV’s extrahepatic manifestations and support risk stratification in clinical practice.

In this study, we aimed to evaluate the associations between chronic HBV infection and serum markers of thyroid function (TSH, FT4), systemic inflammation (CRP), and lipid metabolism (HDL, LDL, triglycerides) in a well-characterized adult cohort. We further explored whether these associations varied by HBV viral load, age, and sex. Our findings contribute to a growing body of evidence suggesting that chronic HBV infection may exert subtle but clinically relevant effects beyond the liver, with potential implications for long-term metabolic and endocrine monitoring.

## 2. Materials and Methods

### 2.1. Study Design and Grouping

This retrospective study included 186 individuals who underwent routine medical examinations at Dankook University Hospital, located in Cheonan, South Korea, between 1 June and 31 December 2024. Individuals who underwent HBsAg and HBV DNA testing were included in the analysis. The study protocol was reviewed and approved by the Institutional Review Board of Dankook University (IRB Certificate No. 2023-01-005). As this is a retrospective study, the requirement for informed consent was waived. All procedures were conducted in accordance with the ethical principles outlined in the Declaration of Helsinki.

**Figure 1:**
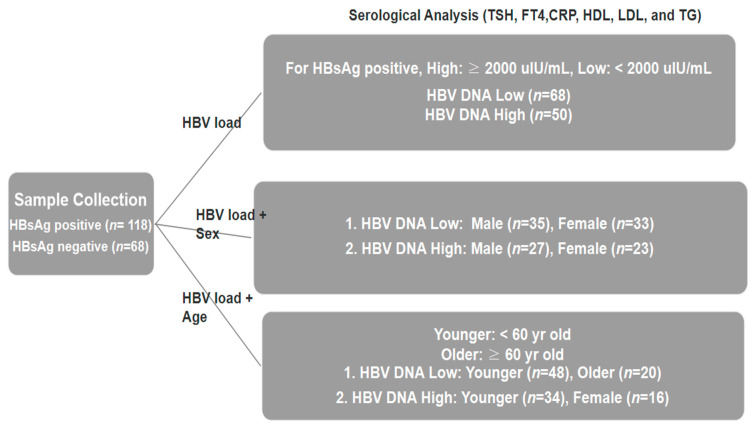
Study design and the number of participants enrolled in the study.

This study included 186 adults: 118 HBsAg-positive and 68 HBsAg-negative controls. HBV positivity was defined by HBsAg seropositivity. Serum biomarkers assessed included TSH, FT4, CRP, HDL cholesterol, LDL cholesterol, and triglycerides to explore HBV-related alterations in thyroid function, lipid metabolism, and inflammation. HBsAg-positive individuals were stratified by serum HBV DNA levels into low (<2000 IU/mL, *n* = 68)- and high (≥2000 IU/mL, *n* = 50)-viral-load groups per international guidelines [18,19,20]. Further stratification by sex (low: 35 Male/33 Female; high: 27 Male/23 Female) and age (<60 vs. ≥60 years) was performed [21]. In the low-viral-load group, 48 were <60 and 20 were ≥60; in the high-viral-load group, 34 were <60 and 16 were ≥60. The HBV-negative group (*n* = 68) served as the reference and was not further stratified (Figure 1).

### 2.2. HBV DNA Measurements

Serum HBV DNA levels were measured using the cobas^®^ HBV test (Roche Diagnostics, Mannheim, Germany), a real-time polymerase chain reaction (PCR) assay on the fully automated cobas^®^ 6800/8800 Systems. Targeting conserved HBV genomic regions (genotypes A–H), the assay offers high specificity and a dynamic range of 10–1.0 × 10^9^ IU/mL, with results standardized to WHO IU/mL. The lower limit of detection is 10 IU/mL. Automated extraction, amplification, and detection reduce contamination risk, while internal and external quality controls ensured accuracy and reproducibility [19,21]. HBV-positive individuals were stratified into low (<2000 IU/mL) and high (≥2000 IU/mL)-viral-load groups based on clinical guidelines to evaluate potential dose-dependent effects on systemic metabolism [20,21,22].

### 2.3. CRP, TSH, and FT4 Measurements

Serum TSH and FT4 were measured via electrochemiluminescence immunoassays (ECLIA) on the Roche Elecsys^®^ e801 analyzer (Roche Diagnostics, Mannheim, Germany), using a sandwich method for TSH and a competitive assay for FT4. Analytical ranges were 0.005–100 μIU/mL (TSH) and 0.023–7.77 ng/dL (FT4), with reference intervals of 0.27–4.20 μIU/mL and 0.93–1.70 ng/dL, respectively [23]. Serum CRP was assessed using a high-sensitivity immunoturbidimetric assay on the cobas^®^ c702 analyzer, based on latex-enhanced immunoagglutination, with a range of 0.3–350 mg/L and reference <0.50 mg/dL. All analyses were performed in a certified lab with strict quality controls [24].

### 2.4. HDL Cholesterol, LDL Cholesterol, and Triglyceride Measurements

Serum HDL cholesterol, LDL cholesterol, and triglycerides were measured using enzymatic colorimetric assays on the cobas^®^ c702 analyzer (Roche Diagnostics, Mannheim, Germany), following the manufacturer’s protocols. HDL cholesterol was assessed via a homogeneous enzymatic method (reference: 40–60 mg/dL), and LDL cholesterol via a direct enzymatic assay (optimal: <130 mg/dL). Triglycerides were classified as <150 (normal), 150–199 (borderline high), 200–499 (high), and ≥500 mg/dL (very high). All tests were conducted under rigorous quality control procedures and interpreted according to international lipid classification guidelines [25].

### 2.5. Statistical Analysis

Continuous variables, assumed to follow a normal distribution, are expressed as mean ± standard deviation. Group differences were evaluated using one-way analysis of variance (ANOVA) followed by Bonferroni post hoc adjustments to account for multiple comparisons. It should be noted that these analyses did not adjust for potential confounders such as age, sex, or comorbidities, as no covariate-adjusted methods (e.g., ANCOVA or multivariable regression) were applied at this stage. Statistical analyses were conducted using GraphPad Prism (version 7.00.159, Dotmatics, Boston, MA, USA), with statistical significance defined as *p* < 0.05. Subsequently, to investigate the independent and interactive effects of HBV DNA levels, age, and sex on serum concentrations of TSH, FT4, CRP, and lipid parameters, multiple linear regression and analysis of covariance (ANCOVA) were performed utilizing Jamovi software (version 2.6.26, The Jamovi project, Australia).

## 3. Results

### 3.1. Comparative Analysis of Thyroid, Inflammatory, and Lipid Biomarkers Between HBsAg-Negative and -Positive Individuals

**Figure 2:**
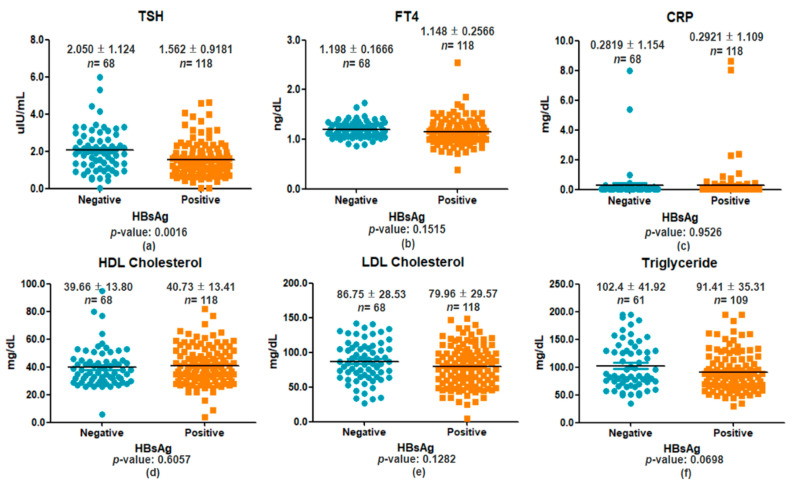
Comparison of serum biomarkers between HBsAg-negative and -positive individuals. Serum levels of TSH, FT4, and CRP (**a**–**c**) and HDL cholesterol, LDL cholesterol, and Triglycerides (**d**–**f**). Data are presented as mean ± standard deviation. Individual values are plotted (HBsAg-negative: blue circles; HBsAg-positive: orange squares). Group sizes were *n* = 68 for HBsAg-negative and *n* = 118 for HBsAg-positive individuals. Statistical comparisons were performed using unpaired two-tailed t-tests, with significance set at *p* < 0.05.

To assess the impact of HBV infection on thyroid function and inflammation, serum TSH, FT4, and CRP levels were compared between HBsAg-negative and -positive individuals. TSH was significantly lower in the HBsAg-positive group (1.562 ± 0.9181 µIU/mL, *n* = 118) than in the negative group (2.050 ± 1.124 µIU/mL, *n* = 68; *p* = 0.0016; Figure 2a), while FT4 (*p* = 0.1515; Figure 2b) and CRP (*p* = 0.9526; Figure 2c) showed no significant differences. Lipid profiles showed no significant group differences in HDL (*p* = 0.6057; Figure 2d), LDL (*p* = 0.1282; Figure 2e), or triglycerides (*p* = 0.0698; Figure 2f). These findings suggest HBV infection is associated with TSH suppression, independent of FT4, CRP, or lipid alterations.

### 3.2. Comparison of Thyroid Function, Inflammatory, and Lipid Markers Among HBsAg-Negative, HBV DNA-Low, and HBV DNA-High Groups

**Figure 3:**
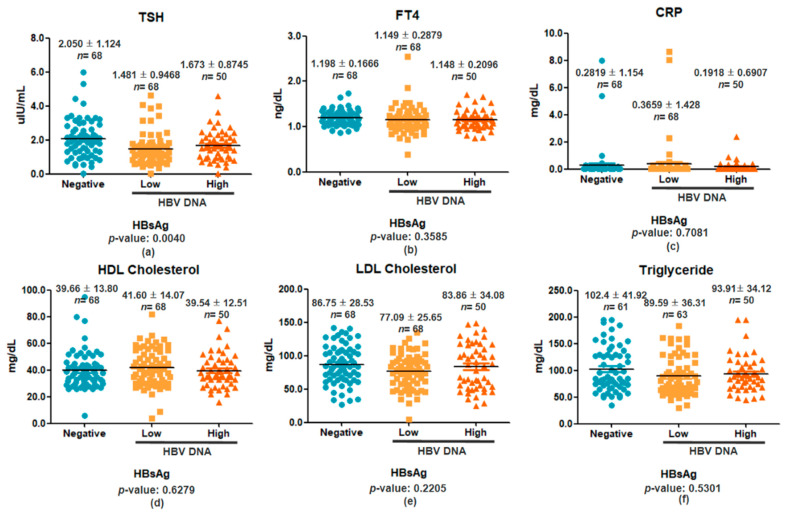
Comparison of serum biomarker levels among individuals stratified by HBsAg status: Negative, Positive Low (<2000 IU/mL), and Positive High (≥2000 IU/mL). Panels (**a**–**c**) show serum levels of TSH, FT4, and CRP, respectively, while panels (**d**–**f**) present HDL, LDL, and triglyceride levels. Data are presented as mean ± SD, with individual data points overlaid. The number of participants (*n*) and mean ± SD values are shown above each group. Statistical analysis was performed using one-way ANOVA followed by Bonferroni post hoc tests to assess pairwise group differences.

Figure 3 presents serum TSH, FT4, and CRP concentrations stratified by HBsAg status: Negative, HBV DNA Low, and HBV DNA High. An initial overall test showed a significant difference in TSH levels among groups (*p* = 0.0040), with both HBsAg-positive subgroups exhibiting lower TSH compared to the HBsAg-negative group. However, pairwise comparisons between the HBsAg-negative group and each positive subgroup revealed no statistically significant differences in TSH (*p* = 0.2617), FT4 (*p* = 0.9888), or CRP (*p* = 0.4017) (Figure 3a–c). This suggests that the initial association between HBsAg positivity and TSH reduction does not hold when accounting for viral-load stratification. Serum lipid profiles, analyzed by one-way ANOVA with Bonferroni corrections, showed no significant differences among HBsAg-negative, HBV DNA Low, and HBV DNA High groups in HDL cholesterol (*p* = 0.6279), LDL cholesterol (*p* = 0.2205), or triglycerides (*p* = 0.5301) (Figure 3d–f). These findings indicate that neither HBsAg status nor viral load significantly impact serum lipid or inflammatory markers in this cohort. Thus, while the overall comparison suggested lower TSH in HBsAg-positive individuals, this effect diminished upon stratification by viral load, indicating that the association may be context-dependent.

Overall ANOVA suggested a group effect on TSH, but only the Negative vs. Low comparison remained significant after correction, and Negative vs. High was not significant. Hence, the overall association diminished upon viral-load stratification. No significant differences were found in FT4, CRP, HDL, LDL, or triglycerides across groups (Supplementary Table S1).

### 3.3. Inflammatory, Thyroid Hormone and Lipid Levels in Relation to Sex and HBV DNA Status

**Figure 4:**
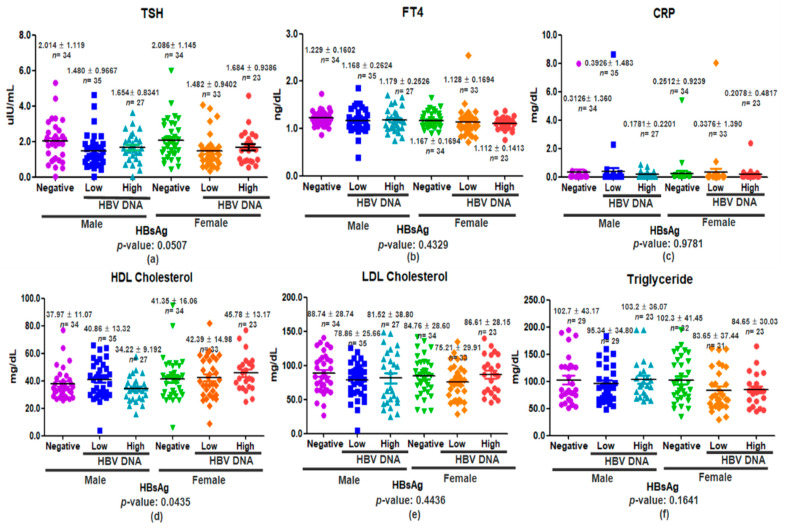
Serum levels of TSH, FT4, CRP and lipid profiles in male and female participants stratified by HBV status. Scatter plots represent individual values of (**a**) TSH, (**b**) FT4, (**c**) CRP, (**d**) HDL cholesterol, (**e**) LDL cholesterol, and (**f**) triglycerides across HBV categories (HBsAg-Negative, HBV DNA Low: <2000 IU/mL, and HBV DNA High: ≥2000 IU/mL) in male and female groups. Horizontal black bars indicate the group means ± standard deviation. The number of participants (*n*) and mean ± SD are displayed above each group. Statistical analysis was conducted using one-way ANOVA followed by Bonferroni post hoc tests to assess pairwise differences.

Serum TSH, FT4, and CRP levels were analyzed by HBsAg status—Negative, HBV DNA Low (<2000 IU/mL), and HBV DNA High (≥2000 IU/mL)—with sex-specific assessment. TSH was highest in HBsAg-negative individuals for both sexes, showing a borderline significant difference between groups (*p* = 0.0507; Figure 4a). FT4 levels varied little and were not significantly associated with HBV status (*p* = 0.4329; Figure 4b). CRP showed no significant group differences (*p* = 0.9781; Figure 4c). These results indicate a possible inverse link between HBV DNA and TSH, without effects on FT4 or CRP, regardless of sex.

**Figure 5:**
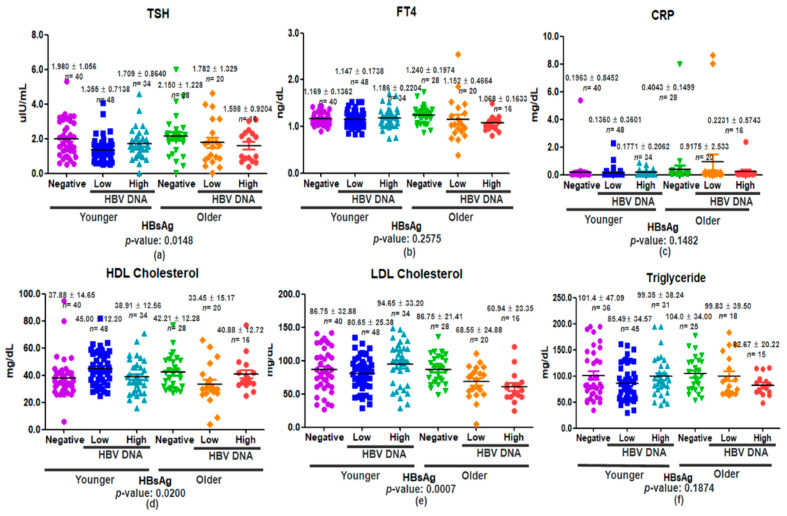
Serum levels of TSH (**a**), FT4 (**b**), CRP (**c**), HDL cholesterol (**d**), LDL cholesterol (**e**), and triglycerides (**f**) in individuals stratified by age and HBsAg status. Participants were grouped into younger (<60 years) and older (≥60 years) categories, and HBsAg status was classified as negative, low (<2000 IU/mL), or high (≥2000 IU/mL). Horizontal black bars represent mean ± standard deviation for each group. Mean values, sample sizes (*n*), and standard deviations are indicated above each group. Statistical comparisons were performed using one-way ANOVA, with *p*-values provided below each panel.

Serum lipid profiles were analyzed by HBsAg status and sex. HDL cholesterol differed significantly between HBV groups (*p* = 0.0435; Figure 4d), with males showing highest HDL at low viral load and females exhibiting progressively higher HDL with increasing viral load. LDL cholesterol showed no significant sex-specific differences across HBV categories (*p* = 0.4436; Figure 4e), nor did triglycerides (*p* = 0.1641; Figure 4f). These results suggest modest sex-specific effects of HBV on HDL, while LDL and triglycerides remain unaffected by HBsAg status alone. Age-stratified analyses (Figure 5e) indicate age may more strongly modify LDL responses to HBV than sex.

Bonferroni-corrected pairwise comparisons showed that males with high HBV DNA had significantly lower HDL cholesterol than females (mean difference = −11.56 mg/dL, *p* < 0.05, 95% CI: −22.78 to −0.34). No other significant differences were found for HDL, LDL, or triglycerides across HBsAg groups by sex (*p* > 0.05). Similarly, TSH, FT4, and CRP levels did not differ significantly between HBsAg-negative and HBV DNA Low/High groups in either sex after correction (*p* > 0.05) (Supplementary Table S2).

### 3.4. Inflammatory, Thyroid Hormone and Lipid Levels in Relation to Age and HBV DNA Status

Serum concentrations of TSH, FT4, and CRP were analyzed according to HBsAg status—categorized as negative, low (<2000 IU/mL), or high (≥2000 IU/mL)—in participants stratified by age (<60 years vs. ≥60 years). When stratified by age, the most notable difference was observed between younger (<60 years) individuals with low HBV DNA levels and older (≥60 years) HBV-negative individuals, with the younger group exhibiting significantly lower TSH concentrations (mean difference: −0.79, *p* = 0.0035; Supplementary Table S3). No other age-based pairwise comparisons reached statistical significance, suggesting that age-dependent TSH suppression is particularly associated with low-level HBV replication (Figure 5a). In contrast, FT4 levels did not significantly differ by HBsAg status (*p* = 0.2575; Figure 5b). While CRP levels also showed no significant differences (*p* = 0.1482), greater variability and elevated mean values were noted in older individuals, potentially reflecting age-related inflammatory changes (Figure 5c). These observations point toward a possible interaction between HBV status and age in modulating thyroid function, particularly with respect to TSH levels.

Serum lipid profiles (HDL, LDL, triglycerides) were analyzed by HBsAg status and age group (<60 vs. ≥60 years). Serum HDL cholesterol levels varied significantly across HBsAg categories (*p* = 0.0200), with the strongest pattern observed in the younger (<60 years) subgroup. In this group, HDL was highest in the HBV DNA–High category (67.3 ± 15.2 mg/dL), followed by HBV DNA–Low (60.9 ± 14.1 mg/dL) and HBsAg-negative (55.8 ± 13.4 mg/dL) participants. The difference between HBV DNA–High and HBsAg-negative groups was significant (+11.47 mg/dL, *p* = 0.0072), whereas no significant differences were detected in the ≥60 years subgroup. These results suggest that high-level HBV replication is associated with increased HDL cholesterol, particularly in younger adults. Cross-age comparisons were avoided to reduce potential confounding by age-related lipid differences (*p* = 0.0007; Figure 5e). When stratified by age, LDL cholesterol was significantly higher in younger individuals with high HBV DNA levels (118.6 ± 32.4 mg/dL) than in their HBsAg-negative counterparts (101.2 ± 29.8 mg/dL; *p* < 0.001). In contrast, among older participants, LDL levels were slightly lower in the HBV DNA–High group (105.7 ± 30.5 mg/dL) than in the HBsAg-negative group (110.2 ± 31.1 mg/dL), but this difference was not statistically significant (*p* = 0.412) (Supplementary Table S3, Figure 5e). These results indicate that the association between HBV replication and LDL cholesterol elevation is prominent only in younger adults. Triglycerides showed no significant differences (*p* = 0.1874), though HBsAg-negative individuals had slightly higher means (Figure 5f). These results indicate age-modulated effects of HBV on lipid metabolism, with younger individuals more susceptible to LDL elevation under high viral load.

Bonferroni-corrected pairwise comparisons revealed significant differences in serum TSH, HDL cholesterol, and LDL cholesterol levels across age groups and HBsAg status. TSH levels were significantly higher in older HBsAg-negative participants compared to younger individuals with low HBV DNA levels (*p* < 0.05). HDL cholesterol was elevated in younger participants with low HBV DNA levels relative to their older counterparts in the same HBV category (*p* < 0.05). LDL cholesterol levels were significantly elevated in younger individuals with high HBV DNA levels compared to their older counterparts in both the low and high viral-load groups (*p* < 0.05, *p* < 0.01), suggesting an age-dependent susceptibility to LDL elevation in the context of HBV antigenemia. No statistically significant differences were observed for FT4, CRP, or triglyceride levels (Supplementary Table S3).

### 3.5. Multivariate Assessment of the Impact of HBV DNA Level, Age, and Sex on TSH, FT4, CRP and Lipid Profiles Using Multiple Linear Regression and ANCOVA

To assess the independent and interactive effects of HBV DNA levels, age, and sex on TSH, FT4, CRP, and lipid profiles, we used multiple linear regression and ANCOVA. Age emerged as the only significant predictor of TSH (β = 1.28, *p* < 0.001), with older individuals showed higher TSH levels; sex and HBV DNA were non-significant. ANCOVA confirmed age as the sole influencing factor (F(1,110) = 76.65, *p* < 0.001), with no significant interactions (Supplementary Table S4). FT4 was not significantly associated with any predictor (all *p* > 0.28), and model fit was low (R^2^ = 0.0431); ANCOVA results were consistent (Supplementary Table S5). CRP showed a negative association with age (β = −0.4613, *p* = 0.038), while sex and HBV DNA had no effect; ANCOVA indicated a marginal age effect (F = 3.51, *p* = 0.064) and no significant interactions (Supplementary Table S6). HDL cholesterol was significantly associated with sex (β = −5.76, *p* = 0.018) and age (β = 5.65, *p* = 0.031), but not HBV DNA. ANCOVA revealed significant effects of age (F = 4.72, *p* = 0.032) and three interactions: sex × HBV DNA (F = 4.26, *p* = 0.041), sex × age (F = 5.05, *p* = 0.027), and HBV DNA × age (F = 6.74, *p* = 0.011), suggesting context-specific modulation (Supplementary Table S7). LDL cholesterol was significantly influenced by age (β = 21.50, *p* < 0.001); other predictors were non-significant. ANCOVA confirmed a strong age effect (F = 16.74, *p* < 0.001), with a borderline HBV DNA × age interaction (F = 3.74, *p* = 0.056) (Supplementary Table S8). For triglycerides, sex was the only significant predictor (β = 14.572, *p* = 0.032), with higher levels in males. ANCOVA revealed a significant HBV DNA × age interaction (F = 4.14, *p* = 0.045), suggesting age-related viral effects (Supplementary Table S9).

## 4. Discussion

This study examined the extrahepatic effects of chronic HBV infection on thyroid function, inflammation, and lipid metabolism. TSH levels were significantly lower in HBsAg-positive individuals compared with controls. However, when stratified by viral load, pairwise differences were attenuated, suggesting that the overall association may not be fully independent of HBV DNA levels. FT4 and CRP were unaffected. Before interpreting lipid-related findings, we note that key covariates were unavailable in this retrospective dataset, including adiposity and metabolic status (e.g., BMI, HOMA-IR), liver injury and disease activity markers (ALT/AST and fibrosis stage), HBV phase markers (e.g., HBeAg), and medication use (e.g., statins, thyroid agents), as well as major comorbidities (e.g., diabetes, metabolic syndrome). These omissions raise the possibility of residual confounding and warrant a conservative interpretation of subgroup lipid patterns in relation to HBV [26].

TSH suppression was observed in HBsAg-positive individuals compared with controls. However, this effect was attenuated when stratifying by viral load, suggesting that antigenemia rather than replication intensity may play a role, although the independence from viral load was less clear in subgroup analyses. This aligns with evidence that HBV affects thyroid function through mechanisms beyond viral burden. Although TSH levels were significantly reduced in HBV-positive individuals, all values were within the clinical reference range (0.27–4.20 µIU/mL), indicating no overt thyroid dysfunction. This pattern suggests a subclinical modulation of the HPT axis—potentially immune-mediated—rather than a consequence of peripheral thyroid hormone deficiency. Importantly, higher HBV DNA or HBsAg levels do not imply a proportional increase in expression of other viral proteins with direct cytopathic effects. HBV replication itself is not directly cytopathic, and disease severity reflects a multifactorial interplay dominated by host immune-mediated injury (e.g., cytokine-driven inflammation, oxidative and endoplasmic reticulum stress) together with contributions from specific viral components and genotypes; thus, antigenemia and viral load are imperfect surrogates for pathogenic protein activity [26].

Age- and sex-specific differences in thyroid profiles among HBV-infected individuals suggest a host-modulated endocrine response. Younger adults (<60 years) show lower TSH levels than older adults (≥60 years), indicating age-related modulation [27]. Sex-stratified analyses further support demographic influences on thyroid function in viral hepatitis [28]. Additionally, HBV treatments such as pegylated interferon-α have been linked to thyroid dysfunction in 8.8% of cases, typically resolving post-therapy [29], underscoring both viral and iatrogenic effects on thyroid regulation.

Our findings extend prior evidence by showing that TSH suppression occurs in HBsAg-positive individuals even without cirrhosis, thyroid disease, or antiviral treatment. TSH levels were significantly lower in HBsAg-positive vs. negative individuals, yet unaffected by viral load (<2000 vs. ≥2000 IU/mL), suggesting that HBV antigens (HBsAg, HBeAg) may alter HPT axis regulation via immune or hepatic-endocrine pathways, independent of replication intensity [27,28,29].

Mechanistically, HBsAg and related antigens may affect hypothalamic–pituitary function via immunomodulatory pathways, including cytokine-mediated crosstalk or molecular mimicry. HBV DNAemia can activate IL-6 or TNF-α, which suppress TRH and/or TSH secretion. Studies in pregnant women with chronic HBV found no correlation between HBV DNA and TSH, but a negative association with FT3 and FT4, suggesting replication intensity impacts peripheral hormone metabolism, while TSH is influenced by antigenic or cytokine-driven central mechanisms [29,30,31,32].

Despite reduced TSH, FT4 levels remained stable, suggesting a centrally mediated, possibly subclinical, adaptation of the HPT axis. This pattern may reflect a compensatory state or an altered set-point driven by chronic viral antigen exposure [32,33].

In this cohort, CRP levels showed no significant association with HBV status or viral load, consistent with previous studies indicating that low-grade systemic inflammation is not characteristic of early or inactive HBV infection [16,34]. While prior reports have suggested CRP elevation in advanced liver disease stages such as cirrhosis or HCC, our findings indicate that CRP is not a useful biomarker of viral activity in the early phase [34,35]. Future longitudinal studies incorporating advanced disease cohorts are warranted to clarify its potential role [33,35,36,37].

This study examined the relationship between HBV DNA status and lipid profiles (HDL, LDL, triglycerides). Although no significant differences were observed across HBsAg-negative, low (<2000 IU/mL), and high (≥2000 IU/mL) HBV groups in the overall cohort, age- and sex-stratified analyses revealed notable subgroup-specific trends. Taken together with the absence of BMI, liver enzymes, fibrosis staging, and medication/comorbidity data, these lipid signals should be viewed as subgroup-specific trends rather than population-wide effects. Unmeasured metabolic status or treatment exposures could plausibly account for part of the observed HDL/LDL differences, limiting causal attribution to HBV per se.

HDL cholesterol levels showed a significant interaction between sex and HBsAg status. Males with high HBV DNA had notably lower HDL levels than females in the same category, suggesting sex-specific effects of HBV on reverse cholesterol transport. Prior studies reported reduced HDL in HBV-infected individuals [38]; our results extend this by revealing sex-based differences. Estrogens promote HDL production via apolipoprotein A1 and ABCA1 upregulation, while androgens may inhibit these pathways [39], possibly explaining the observed disparity.

LDL cholesterol was elevated in younger individuals with high HBV DNA levels when compared to age-matched controls, suggesting that viral replication may have a greater impact on LDL metabolism in younger adults [40]. We avoided direct comparisons across different age groups (e.g., younger HBsAg-negative vs. older HBV-positive) due to potential confounding by age-related lipid metabolism changes. This may involve HBV-mediated interference with transcriptional regulators like SREBPs [41], and viral proteins such as HBx affecting HNF4α and PPARs, key factors in lipid transport and storage [42].

Triglyceride levels did not differ significantly by HBV status in our cohort, partly aligning with prior reports of inverse associations [43,44]. This may reflect cohort-specific factors or confounders. Mechanistically, lower triglycerides in HBV-infected individuals have been linked to downregulation of lipogenesis and VLDL secretion genes, along with inflammation-driven metabolic changes [45].

In this study, no significant differences in lipid parameters were observed in the overall cohort. However, subgroup analyses revealed age- and sex-specific patterns, such as lower HDL in males with high viral load and higher LDL in younger HBV-positive adults. These findings suggest that potential HBV-related effects on lipid metabolism are restricted to certain demographic subgroups rather than universal across all patients. These effects likely stem from HBV-driven changes in hepatocellular transcription, lipid biosynthesis, and inflammation. Future research using functional lipidomics and longitudinal data may clarify the metabolic and cardiovascular implications of chronic HBV infection.

Multivariable analyses identified age as a key determinant of TSH, CRP, HDL, and LDL levels across HBV DNA groups. Younger individuals showed higher TSH and lower CRP, consistent with age-related hormonal and inflammatory trends. While HBV DNA alone had no significant biomarker effect, interactions emerged—HDL levels were influenced by HBV DNA, sex, and age, and triglycerides were higher in males with age-specific HBV responses. These findings highlight the importance of demographic context in assessing HBV-related biomarker changes.

Although our study added granularity by integrating viral load, sex, and age into the analysis of endocrine and metabolic parameters, it had key limitations. First, its cross-sectional design precluded causal inference and limited the assessment of temporal or dynamic changes in thyroid, inflammatory, or lipid markers during HBV disease progression. Longitudinal studies demonstrated that lipid and liver function profiles in chronic HBV patients fluctuated over time depending on disease activity and treatment status [46]. Notably, HBV DNA levels were inversely associated with triglyceride levels, with ALT modifying this relationship—suggesting dynamic interactions between viral replication and lipid metabolism [47,48]. These findings underscored the value of longitudinal analyses in capturing the evolving interplay between HBV infection and metabolic regulation.

Second, the absence of key clinical data—such as ALT, AST, HBV genotype, liver fibrosis scores, BMI, insulin resistance (e.g., HOMA-IR), and lifestyle factors—raises the risk of residual confounding [48,49,50]. Due to the retrospective design, liver enzyme data were unavailable, limiting assessment of hepatic inflammation’s role in metabolic and endocrine changes. Prior studies link ALT/AST to dyslipidemia, inflammation, and HBV replication [48,49,50]. Future research should include comprehensive liver function tests to clarify these associations. In particular, the lack of BMI, ALT/AST, fibrosis staging, and medication/comorbidity data constrains interpretation of HDL/LDL findings and may partially explain subgroup differences that otherwise appear to track with HBV activity [50].

Third, virological assessment was limited to HBsAg and HBV DNA, excluding key markers like HBeAg, anti-HBc IgM, and cccDNA [51,52,53,54,55]. These are essential for distinguishing infection phases. HBeAg indicates active replication [51,54], anti-HBc IgM helps differentiate acute from chronic infection [52,54], and cccDNA reflects persistent viral templates in hepatocytes [53,55]. Their absence limits precise classification of HBV phase and related metabolic or endocrine effects.

Fourth, missing data on comorbidities—such as diabetes, metabolic syndrome—and use of lipid- or thyroid-modulating medications may introduce residual confounding [56]. These factors significantly influence lipid and thyroid profiles in CHB patients. Metabolic syndrome and diabetes are linked to dyslipidemia and liver disease progression [56,57], while medications like statins and thyroid agents can independently alter biomarker levels [56,58]. Their absence limits proper adjustment and may bias results. Consequently, residual confounding by these factors cannot be excluded when considering the observed subgroup lipid patterns.

Fifth, subgroup analyses by sex, age, and HBV DNA were limited by small sample sizes, reducing power to detect modest effects and limiting generalizability [59,60,61]. This highlights the need for larger studies to assess demographic interactions with HBV-related metabolic and endocrine changes. Additionally, the cross-sectional design prevents causal inference. Longitudinal research is needed to clarify whether these alterations precede or result from HBV disease activity and to assess their impact on progression and treatment outcomes [59,60,61].

Sixth, unlike prior studies that assessed single biomarkers, our study integrated thyroid, lipid, and inflammatory profiles within one HBV cohort, offering a more comprehensive view. Building on evidence that HBV acts as a “metabolic virus” altering hepatic lipid pathways [61], we performed subgroup analyses by age, sex, and HBV DNA—rarely combined in previous work. This revealed context-specific patterns, such as sex-based HDL differences at high viral load and age-related CRP trends independent of replication. Using quantitative HBV DNA rather than binary antigen status allowed finer stratification of viral activity. Our findings suggest that antigenemia and host demographics jointly shape extrahepatic responses, especially in early or inactive disease, informing personalized monitoring strategies.

Seventh, while our study offers novel insights into HBV’s extrahepatic effects, the absence of critical clinical variables—such as liver enzymes, fibrosis stage, and BMI—limits interpretive precision. These factors influence inflammation and lipid metabolism, introducing potential confounding. For instance, BMI strongly affects lipid profiles, and liver dysfunction can independently disrupt cholesterol regulation. Incorporating these parameters in future studies will be essential to disentangle the effects of HBV replication and antigenemia from host metabolic status, particularly in distinguishing viral activity from hepatic injury in shaping thyroid and lipid outcomes [62].

Despite its limitations, this study adds to evidence that chronic HBV infection influences extrahepatic systems, particularly endocrine and lipid regulation. The observed TSH suppression, along with age- and sex-specific shifts in HDL and LDL levels, highlights the need for metabolic surveillance in HBV-infected individuals. Given the rising concern over metabolic syndrome and cardiovascular risk in chronic liver disease, these findings may inform clinical risk stratification. Future prospective studies incorporating liver function data, hormone panels, and mechanistic biomarkers are needed to clarify the pathways linking HBV to systemic metabolic dysregulation.

## 5. Conclusions

Chronic HBV infection is associated with subtle alterations in thyroid function and lipid metabolism that are modulated by age and sex, and may be more closely related to antigenemia than replication intensity, although subgroup analyses showed attenuation with viral-load stratification. TSH suppression occurred in HBsAg-positive individuals without exceeding clinical reference limits, suggesting a subclinical modulation of the hypothalamic–pituitary–thyroid axis. Lipid changes were context-specific, with lower HDL in males with high viral load and higher LDL in younger HBV-positive adults, while CRP remained unaffected in early or inactive disease. These patterns highlight the importance of incorporating viral, demographic, and metabolic variables into the clinical assessment of HBV-infected patients. Longitudinal studies including comprehensive virological and metabolic markers are warranted to elucidate causal mechanisms and inform strategies to mitigate cardiovascular and metabolic risk.

## Data Availability

The datasets utilized and examined in this study can be obtained from the corresponding author upon request deemed reasonable.

## Supplementary Materials

The following supporting information can be downloaded at: https://www.mdpi.com/article/10.3390/v17091208/s1, Table S1: Statistical analysis of inflammatory, thyroid, and lipid parameters according to HBV antigen status using Bonferroni’s test; Table S2: Pairwise comparisons of serum TSH, FT4, CRP, HDL cholesterol, LDL cholesterol, and triglyceride levels among male and female participants stratified by HBV status; Table S3: Pairwise comparisons of serum TSH, FT4, CRP, HDL cholesterol, LDL cholesterol, and triglyceride levels among younger (<60 yr old) and elder (≥60 yr old) participants stratified by HBV status; Table S4: Associations of age, sex, and HBV DNA status with serum TSH concentrations: results from multiple linear regression and ANCOVA models; Table S5: Associations of age, sex, and HBV DNA status with serum FT4 concentrations: results from multiple linear regression and ANCOVA models; Table S6: Associations of age, sex, and HBV DNA status with serum CRP concentrations: results from multiple linear regression and ANCOVA models; Table S7: Associations of age, sex, and HBV DNA status with serum HDL Cholesterol concentrations: results from multiple linear regression and ANCOVA models; Table S8: Associations of age, sex, and HBV DNA status with serum LDL Cholesterol concentrations: results from multiple linear regression and ANCOVA models; Table S9: Associations of age, sex, and HBV DNA status with serum Triglyceride concentrations: results from multiple linear regression and ANCOVA models.

## Abbreviations

ALT	Alanine aminotransferase
ANCOVA	Analysis of covariance
AST	Aspartate aminotransferase
BMI	Body mass index
cccDNA	Covalently closed circular DNA
CHB	Chronic hepatitis B
CRP	C-reactive protein
ECLIA	Electrochemiluminescence immunoassay
FT4	Free thyroxine
HBsAg	Hepatitis B surface antigen
HBV	Hepatitis B virus
HDL	High-density lipoprotein
HOMA-IR	Homeostatic model assessment of insulin resistance
HPT axis	Hypothalamic–pituitary–thyroid axis
LDL	Low-density lipoprotein
PCR	Polymerase chain reaction
SREBP	Sterol regulatory element-binding protein
TG	Triglycerides
TRH	Thyrotropin-releasing hormone
TSH	Thyroid-stimulating hormone
