# Supplementary material for: Multivariate Assessment of Thyroid, Lipid, and Inflammatory Profiles by HBV Status and Viral Load: Age- and Sex-Specific Findings
Source: Viruses. 2025 Sep 3;17(9):1208. doi: 10.3390/v17091208 (PMC12474459; doi:10.3390/v17091208)
Supplement: Supplementary file 1 [file viruses-17-01208-s001.zip › viruses-3845661-supplementary.pdf]

Table S1. Statistical analysis of inflammatory, thyroid, and lipid parameters according to HBV antigen status using Bonferroni's test (NS: Not Significant, CI: Confidence Interval). Pairwise comparisons were conducted among HBV-negative, HBV-positive low (<2000 IU/mL), and HBV-positive high (≥2000 IU/mL) groups. Results include mean differences, p-values, significance status (P < 0.05), and corresponding 95% confidence intervals (CIs). A significant difference was observed only in TSH levels between the HBV-negative and HBV-positive low groups. No significant differences were detected in FT4, CRP, HDL cholesterol, LDL cholesterol, or triglyceride levels across the groups.

**TSH**	Mean Diff.	*t*	*P* < 0.05?	Summary	95% CI of diff
Negative vs. Positive Low	0.5693	3.326	Yes	**	0.1558 to 0.9828
Negative vs Positive High	0.3766	2.026	No	**NS**	-0.07258 to 0.8258
Positive Low vs. Positive High	-0.1927	1.036	No	**NS**	-0.6418 to 0.2565
**FT4**	Mean Diff.	*t*	*P* < 0.05?	Summary	95% CI of diff
Negative vs. Positive Low	0.04971	1.268	No	**NS**	-0.1564 to 0.1331
Negative vs Positive High	0.05038	1.183	No	**NS**	-0.07207 to 0.2164
Positive Low vs. Positive High	0.00067	0.01588	No	**NS**	-0.1435 to 0.1008
**CRP**	Mean Diff.	*t*	*P* < 0.05?	Summary	95% CI of diff
Negative vs. Positive Low	-0.08397	0.4347	No	**NS**	-0.5507 to 0.3828
Negative vs Positive High	0.09011	0.4294	No	**NS**	-0.4169 to 0.5971
Positive Low vs. Positive High	0.1741	0.8296	No	**NS**	-0.3329 to 0.6811
**HDL Cholesterol**	Mean Diff.	*t*	*P* < 0.05?	Summary	95% CI of diff
Negative vs. Positive Low	-1.941	0.8344	No	**NS**	-7.562 to 3.680
Negative vs Positive High	0.1218	0.04818	No	**NS**	-5.985 to 6.228
Positive Low vs. Positive High	2.063	0.8163	No	**NS**	-4.043 to 8.169
**LDL Cholesterol**	Mean Diff.	*t*	*P* < 0.05?	Summary	95% CI of diff
Negative vs. Positive Low	9.662	1.932	No	**NS**	-2.418 to 21.74
Negative vs Positive High	2.890	0.5321	No	**NS**	-10.23 to 16.01
Positive Low vs. Positive High	-6.772	1.247	No	**NS**	-19.89 to 6.351
**Triglyceride**	Mean Diff.	*t*	*P* < 0.05?	Summary	95% CI of diff
Negative vs. Positive Low	12.86	1.889	No	**NS**	-3.599 to 29.31
Negative vs Positive High	8.530	1.153	No	**NS**	-9.359 to 26.42
Positive Low vs. Positive High	-4.326	0.5888	No	**NS**	-22.09 to 13.44

*p < 0.05, **p < 0.01, *** p <0.001, **NS**: Not Significant.

Table S2. Pairwise comparisons of serum TSH, FT4, CRP, HDL cholesterol, LDL cholesterol, and triglyceride levels among male and female participants stratified by HBV status. Mean differences, t-values, p-values, significance summaries, and 95% confidence intervals (CIs) are shown for each comparison. Group abbreviations: M. = Male; F. = Female; (−) = HBV negative; (+) Low = HBV DNA < 2000 IU/mL; (+) High = HBV DNA ≥ 2000 IU/mL. Statistical significance was assessed using one-way ANOVA followed by Bonferroni-corrected post hoc multiple comparisons.

**TSH**	Mean Diff.	*t*	*P* < 0.05?	Summary	95% CI of diff
M. (-) vs. M. (+) Low	0.5344	2.206	No	**NS**	-0.1862 to 1.255
M. (-) vs. M. (+) High	0.3503	1.351	No	**NS**	-0.4211 to 1.122
M. (-) vs. F. (-)	-0.7118	0.2917	No	**NS**	-0.7970 to 0.6546
M. (-) vs. F. (+) Low	0.5329	2.168	No	**NS**	-0.1984 to 1.264
M. (-) vs. F. (+) High	0.3301	1.215	No	**NS**	-0.4779 to 1.138
M. (+) Low vs. M. (+) High	-0.1841	0.7144	No	**NS**	-0.9506 to 0.5824
M. (+) Low vs. F. (-)	-0.6056	2.500	No	**NS**	-1.326 to 0.1150
M. (+) Low vs. F. (+) Low	-0.001515	0.006208	No	**NS**	-0.7276 to 0.7246
M. (+) Low vs. F. (+) High	-0.2043	0.7568	No	**NS**	-1.008 to 0.5989
M. (+) High vs. F. (-)	-0.4215	1.626	No	**NS**	-1.193 to 0.3499
M. (+) High vs. F. (+) Low	0.1826	0.6994	No	**NS**	-0.5940 to 0.9591
M. (+) High vs. F. (+) High	-0.02027	0.07103	No	**NS**	-0.8694 to 0.8289
F. (-) vs. F. (+) Low	0.6041	2.457	No	**NS**	-0.1272 to 1.335
F. (-) vs. F. (+) High	0.4012	1.477	No	**NS**	-0.4067to 1.209
F. (+) High vs. F. (+) High	-0.2028	0.7423	No	**NS**	-1.016 to 0.6100

**FT4**	Mean Diff.	*t*	*P* < 0.05?	Summary	95% CI of diff
M. (-) vs. M. (+) Low	0.06113	1.11	No	**NS**	-0.1027 to 0.2250
M. (-) vs. M. (+) High	0.05089	0.8632	No	**NS**	-0.1245 to 0.2263
M. (-) vs. F. (-)	0.06206	1.119	No	**NS**	-0.1030 to 0.2271
M. (-) vs. F. (+) Low	0.1015	1.817	No	**NS**	-0.06475 to 0.2678
M. (-) vs. F. (+) High	0.1172	1.898	No	**NS**	-0.06647 to 0.3009
M. (+) Low vs. M. (+) High	-0.01023	0.1747	No	**NS**	-0.1845 to 0.1641
M. (+) Low vs. F. (-)	0.0009328	0.01694	No	**NS**	-0.1629 to 0.1648
M. (+) Low vs. F. (+) Low	0.04041	0.7281	No	**NS**	-0.1247 to 0.2055
M. (+) Low vs. F. (+) High	0.05611	0.9139	No	**NS**	-0.1265 to 0.2388
M. (+) High vs. F. (-)	0.01117	0.1894	No	**NS**	-0.1642 to 0.1866
M. (+) High vs. F. (+) Low	0.05064	0.8532	No	**NS**	-0.1259 to 0.2272
M. (+) High vs. F. (+) High	0.06634	1.022	No	**NS**	-0.1267 to 0.2594
F. (-) vs. F. (+) Low	0.03947	0.7062	No	**NS**	-0.1268 to 0.2058
F. (-) vs. F. (+) High	0.05518	0.8935	No	**NS**	-0.1285 to 0.2389
F. (+) High vs. F. (+) High	0.0157	0.2528	No	**NS**	-0.1691 to 0.2005

**CRP**	Mean Diff.	*t*	*P* < 0.05?	Summary	95% CI of diff
M. (-) vs. M. (+) Low	-0.07992	0.2923	No	**NS**	-0.8933 to 0.7335
M. (-) vs. M. (+) High	0.1345	0.4595	No	**NS**	-0.7362 to 1.005
M. (-) vs. F. (-)	0.06147	0.2232	No	**NS**	-0.7578 to 0.8807
M. (-) vs. F. (+) Low	-0.02493	0.08984	No	**NS**	-0.8504 to 0.8005
M. (-) vs. F. (+) High	0.1048	0.3419	No	**NS**	-0.8072 to 1.017
M. (+) Low vs. M. (+) High	0.2144	0.7372	No	**NS**	-0.6508 to 1.080

M. (+) Low vs. F. (-)	0.1414	0.5171	No	**NS**	-0.6720 to 0.9548
M. (+) Low vs. F. (+) Low	0.055	0.1996	No	**NS**	-0.7646 to 0.8746
M. (+) Low vs. F. (+) High	0.1847	0.6061	No	**NS**	-0.7220 to 1.091
M. (+) High vs. F. (-)	-0.07303	0.2495	No	**NS**	-0.9438 to 0.7977
M. (+) High vs. F. (+) Low	-0.1594	0.5411	No	**NS**	-1.036 to 0.7171
M. (+) High vs. F. (+) High	-0.02968	0.09211	No	**NS**	-0.9882 to 0.9288
F. (-) vs. F. (+) Low	-0.0864	0.3114	No	**NS**	-0.9118 to 0.7390
F. (-) vs. F. (+) High	0.04335	0.1414	No	**NS**	-0.8686 to 0.9553
F. (+) High vs. F. (+) High	0.1297	0.4207	No	**NS**	-0.7878 to 1.047

**HDL Cholesterol**	Mean Diff.	*t*	*P* < 0.05?	Summary	95% CI of diff
M. (-) vs. M. (+) Low	-2.887	0.9021	No	**NS**	-12.41 to 6.632
M. (-) vs. M. (+) High	3.748	1.094	No	**NS**	-6.442 to 13.94
M. (-) vs. F. (-)	-3.382	1.049	No	**NS**	-12.97 to 6.205
M. (-) vs. F. (+) Low	-4.423	1.362	No	**NS**	-14.08 to 5.237
M. (-) vs. F. (+) High	-7.812	2.178	No	**NS**	-18.48 to 2.861
M. (+) Low vs. M. (+) High	6.635	1.949	No	**NS**	-3.491 to 16.76
M. (+) Low vs. F. (-)	-0.4958	0.1549	No	**NS**	-10.01 to 9.023
M. (+) Low vs. F. (+) Low	-1.537	0.4766	No	**NS**	-11.13 to 8.055
M. (+) Low vs. F. (+) High	-4.925	1.381	No	**NS**	-15.54 to 5.685
M. (+) High vs. F. (-)	-7.131	2.082	No	**NS**	-17.32 to 3.059
M. (+) High vs. F. (+) Low	-8.172	2.37	No	**NS**	-18.43 to 2.086
M. (+) High vs. F. (+) High	-11.56	3.066	Yes	*	-22.78 to -0.3435
F. (-) vs. F. (+) Low	-1.041	0.3206	No	**NS**	-10.70 to 8.619
F. (-) vs. F. (+) High	-4.43	1.235	No	**NS**	-15.10 to 6.243
F. (+) High vs. F. (+) High	-3.389	0.9388	No	**NS**	-14.13 to 7.349

**LDL Cholesterol**	Mean Diff.	*t*	*P* < 0.05?	Summary	95% CI of diff
M. (-) vs. M. (+) Low	9.878	1.399	No	**NS**	-11.12 to 30.88
M. (-) vs. M. (+) High	7.217	0.9549	No	**NS**	-15.27 to 29.70
M. (-) vs. F. (-)	3.971	0.5584	No	**NS**	-17.18 to 25.12
M. (-) vs. F. (+) Low	13.52	1.888	No	**NS**	-7.790 to 34.84
M. (-) vs. F. (+) High	2.127	0.2687	No	**NS**	-21.42 to 25.67
M. (+) Low vs. M. (+) High	-2.661	0.3544	No	**NS**	-25.00 to 19.68
M. (+) Low vs. F. (-)	-5.908	0.8368	No	**NS**	-26.91 to 15.09
M. (+) Low vs. F. (+) Low	3.645	0.5124	No	**NS**	-17.52 to 24.81
M. (+) Low vs. F. (+) High	-7.752	0.985	No	**NS**	-31.16 to 15.66
M. (+) High vs. F. (-)	-3.246	0.4295	No	**NS**	-25.73 to 19.24
M. (+) High vs. F. (+) Low	6.306	0.8289	No	**NS**	-16.33 to 28.94
M. (+) High vs. F. (+) High	-5.09	0.6119	No	**NS**	-29.84 to 19.66
F. (-) vs. F. (+) Low	9.553	1.333	No	**NS**	-11.76 to 30.87
F. (-) vs. F. (+) High	-1.844	0.233	No	**NS**	-25.39 to 21.70
F. (+) High vs. F. (+) High	-11.4	1.431	No	**NS**	-35.09 to 12.29

**Triglyceride**	Mean Diff.	*t*	*P* < 0.05?	Summary	95% CI of diff
M. (-) vs. M. (+) Low	7.311	0.7558	No	**NS**	-21.51 to 36.13
M. (-) vs. M. (+) High	-0.5187	0.04924	No	**NS**	-31.90 to 30.87
M. (-) vs. F. (-)	0.4052	0.04188	No	**NS**	-28.41 to 29.22
M. (-) vs. F. (+) Low	19.01	1.95	No	**NS**	-10.03 to 48.05

M. (-) vs. F. (+) High	18	1.709	No	**NS**	-13.38 to 49.39
M. (+) Low vs. M. (+) High	-7.83	0.7591	No	**NS**	-38.56 to 22.90
M. (+) Low vs. F. (-)	-6.906	0.7321	No	**NS**	-35.01 to 21.19
M. (+) Low vs. F. (+) Low	11.7	1.23	No	**NS**	-16.63 to 40.02
M. (+) Low vs. F. (+) High	10.69	1.037	No	**NS**	-20.03 to 41.42
M. (+) High vs. F. (-)	0.9239	0.08957	No	**NS**	-29.80 to 31.65
M. (+) High vs. F. (+) Low	19.53	1.881	No	**NS**	-11.40 to 50.46
M. (+) High vs. F. (+) High	18.52	1.665	No	**NS**	-14.62 to 51.67
F. (-) vs. F. (+) Low	18.6	1.957	No	**NS**	-9.721 to 46.93
F. (-) vs. F. (+) High	17.6	1.706	No	**NS**	-13.13 to 48.32
F. (+) High vs. F. (+) High	-1.007	0.09697	No	**NS**	-31.94 to 29.93

*p < 0.05, **p < 0.01, *** p <0.001, **NS**: Not Significant.

Table S3. Pairwise comparisons of serum TSH, FT4, CRP, HDL cholesterol, LDL cholesterol, and triglyceride levels among younger (< 60 yr old) and elder (≥ 60 yr old) participants stratified by HBV status. Mean differences, t-values, p-values, significance summaries, and 95% confidence intervals (CIs) are shown for each comparison. Group abbreviations: Y. = Younger; E. = Elder; (⁻) = HBV negative; (+) Low = HBV DNA < 2000 IU/mL; (+) High = HBV DNA ≥ 2000 IU/mL. Statistical significance was assessed using one-way ANOVA followed by Bonferroni-corrected post hoc multiple comparisons.

**TSH**	Mean Diff.	*t*	*P* < 0.05?	Summary	95% CI of diff
Y. (-) vs. Y. (+) Low	0.625	2.927	No	**NS**	-0.01017 to 1.260
Y. (-) vs. Y. (+) High	0.2714	1.167	No	**NS**	-0.4207 to 0.9635
Y. (-) vs. E. (-)	-0.1694	0.6893	No	**NS**	-0.9005 to 0.5617
Y. (-) vs. E. (+) Low	0.1983	0.7258	No	**NS**	-0.6143 to 1.011
Y. (-) vs. E. (+) High	0.3821	1.295	No	**NS**	-0.4955 to 1.260
Y. (+) Low vs. Y. (+) High	-0.3536	1.582	No	**NS**	-1.019 to 0.3115
Y. (+) Low vs. E. (-)	-0.7944	3.35	Yes	*	-1.500 to -0.08887
Y. (+) Low vs. E. (+) Low	-0.4268	1.608	No	**NS**	-1.216 to 0.3629
Y. (+) Low vs. E. (+) High	-0.2429	0.8437	No	**NS**	-1.099 to 0.6136
Y. (+) High vs. E. (-)	-0.4408	1.732	No	**NS**	-1.198 to 0.3164
Y. (+) High vs. E. (+) Low	-0.07318	0.2604	No	**NS**	-0.9093 to 0.7629
Y. (+) High vs. E. (+) High	0.1107	0.3661	No	**NS**	-0.7888 to 1.010
E. (-) vs. E. (+) Low	0.3676	1.259	No	**NS**	-0.5010 to 1.236
E. (-) vs. E. (+) High	0.5515	1.764	No	**NS**	-0.3783 to 1.481
E. (+) High vs. E. (+) High	0.1839	0.5497	No	**NS**	-0.8113 to 1.179
**FT4**	Mean Diff.	*t*	*P* < 0.05?	Summary	95% CI of diff
Y. (-) vs. Y. (+) Low	0.02171	0.4454	No	**NS**	-0.1233 to 0.1667
Y. (-) vs. Y. (+) High	-0.01659	0.3123	No	**NS**	-0.1746 to 0.1414
Y. (-) vs. E. (-)	-0.07136	1.272	No	**NS**	-0.2383 to 0.09554
Y. (-) vs. E. (+) Low	0.017	0.2726	No	**NS**	-0.1685 to 0.2025
Y. (-) vs. E. (+) High	0.1009	1.498	No	**NS**	-0.09948 to 0.3012
Y. (+) Low vs. Y. (+) High	-0.0383	0.7504	No	**NS**	-0.1901 to 0.1135
Y. (+) Low vs. E. (-)	-0.09307	1.719	No	**NS**	-0.2541 to 0.06800
Y. (+) Low vs. E. (+) Low	-0.004708	0.0777	No	**NS**	-0.1850 to 0.1756
Y. (+) Low vs. E. (+) High	0.07917	1.205	No	**NS**	-0.1164 to 0.2747
Y. (+) High vs. E. (-)	-0.05477	0.9426	No	**NS**	-0.2276 to 0.1181

Y. (+) High vs. E. (+) Low	0.03359	0.5235	No	**NS**	-0.1573 to 0.2245
Y. (+) High vs. E. (+) High	0.1175	1.702	No	**NS**	-0.08788 to 0.3228
E. (-) vs. E. (+) Low	0.08836	1.326	No	**NS**	-0.1099 to 0.2867
E. (-) vs. E. (+) High	0.1722	2.414	No	**NS**	-0.04003 to 0.3845
E. (+) High vs. E. (+) High	0.08387	1.098	No	**NS**	-0.1433 to 0.3111

**CRP**	Mean Diff.	*t*	*P* < 0.05?	Summary	95% CI of diff
Y. (-) vs. Y. (+) Low	0.06021	0.2528	No	**NS**	-0.6484 to 0.7688
Y. (-) vs. Y. (+) High	0.01919	0.07394	No	**NS**	-0.7529 to 0.7913
Y. (-) vs. E. (-)	-0.208	0.7588	No	**NS**	-1.024 to 0.6076
Y. (-) vs. E. (+) Low	-0.7213	2.367	No	**NS**	-1.628 to 0.1852
Y. (-) vs. E. (+) High	-0.02687	0.08165	No	**NS**	-1.006 to 0.9523
Y. (+) Low vs. Y. (+) High	-0.04102	0.1645	No	**NS**	-0.7830 to 0.7009
Y. (+) Low vs. E. (-)	-0.2682	1.014	No	**NS**	-1.055 to 0.5189
Y. (+) Low vs. E. (+) Low	-0.7815	2.639	No	**NS**	-1.662 to 0.09950
Y. (+) Low vs. E. (+) High	-0.08708	0.2711	No	**NS**	-1.043 to 0.8684
Y. (+) High vs. E. (-)	-0.2272	0.8002	No	**NS**	-1.072 to 0.6175
Y. (+) High vs. E. (+) Low	-0.7404	2.361	No	**NS**	-1.673 to 0.1923
Y. (+) High vs. E. (+) High	-0.04607	0.1366	No	**NS**	-1.050 to 0.9574
E. (-) vs. E. (+) Low	-0.5132	1.575	No	**NS**	-1.482 to 0.4559
E. (-) vs. E. (+) High	0.1812	0.5195	No	**NS**	-0.8562 to 1.219
E. (+) High vs. E. (+) High	0.6944	1.861	No	**NS**	-0.4158 to 1.805

**HDL Cholesterol**	Mean Diff.	*t*	*P* < 0.05?	Summary	95% CI of diff
Y. (-) vs. Y. (+) Low	-7.125	2.518	No	**NS**	-15.54 to 1.293
Y. (-) vs. Y. (+) High	-1.037	0.3363	No	**NS**	-10.21 to 8.135
Y. (-) vs. E. (-)	-4.339	1.332	No	**NS**	-14.03 to 5.349
Y. (-) vs. E. (+) Low	4.425	1.223	No	**NS**	-6.343 to 15.19
Y. (-) vs. E. (+) High	-3	0.7674	No	**NS**	-14.63 to 8.630
Y. (+) Low vs. Y. (+) High	6.088	2.055	No	**NS**	-2.725 to 14.90
Y. (+) Low vs. E. (-)	2.786	0.8864	No	**NS**	-6.564 to 12.14
Y. (+) Low vs. E. (+) Low	11.55	3.284	Yes	*	1.086 to 22.01
Y. (+) Low vs. E. (+) High	4.125	1.081	No	**NS**	-7.225 to 15.48
Y. (+) High vs. E. (-)	-3.303	0.9791	No	**NS**	-13.34 to 6.731
Y. (+) High vs. E. (+) Low	5.462	1.466	No	**NS**	-5.618 to 16.54
Y. (+) High vs. E. (+) High	-1.963	0.49	No	**NS**	-13.88 to 9.957
E. (-) vs. E. (+) Low	8.764	2.265	No	**NS**	-2.747 to 20.28
E. (-) vs. E. (+) High	1.339	0.3233	No	**NS**	-10.98 to 13.66
E. (+) High vs. E. (+) High	-7.425	1.675	No	**NS**	-20.61 to 5.763

**LDL Cholesterol**	Mean Diff.	*t*	*P* < 0.05?	Summary	95% CI of diff
Y. (-) vs. Y. (+) Low	6.104	1.018	No	**NS**	-11.73 to 23.94
Y. (-) vs. Y. (+) High	-7.897	1.209	No	**NS**	-27.33 to 11.53
Y. (-) vs. E. (-)	0	0	No	**NS**	-20.52 to 20.52
Y. (-) vs. E. (+) Low	18.2	2.374	No	**NS**	-4.610 to 41.01
Y. (-) vs. E. (+) High	25.81	3.117	Yes	*	1.175 to 50.45
Y. (+) Low vs. Y. (+) High	-14	2.231	No	**NS**	-32.67 to 4.669
Y. (+) Low vs. E. (-)	-6.104	0.9168	No	**NS**	-25.91 to 13.70
Y. (+) Low vs. E. (+) Low	12.1	1.623	No	**NS**	-10.07 to 34.26

Y. (+) Low vs. E. (+) High	19.71	2.438	No	**NS**	-4.336 to 43.75
Y. (+) High vs. E. (-)	7.897	1.105	No	**NS**	-13.36 to 29.15
Y. (+) High vs. E. (+) Low	26.1	3.308	Yes	*	2.625 to 49.57
Y. (+) High vs. E. (+) High	33.71	3.971	Yes	**	8.458 to 58.96
E. (-) vs. E. (+) Low	18.2	2.22	No	**NS**	-6.185 to 42.59
E. (-) vs. E. (+) High	25.81	2.942	No	**NS**	-0.2903 to 51.92
E. (+) High vs. E. (+) High	7.613	0.8106	No	**NS**	-20.32 to 35.55
**Triglyceride**	Mean Diff.	*t*	*P* < 0.05?	Summary	95% CI of diff
Y. (-) vs. Y. (+) Low	15.87	1.879	No	**NS**	-9.290 to 41.03
Y. (-) vs. Y. (+) High	2.006	0.2167	No	**NS**	-25.57 to 29.58
Y. (-) vs. E. (-)	-2.639	0.2683	No	**NS**	-31.94 to 26.66
Y. (-) vs. E. (+) Low	1.528	0.1401	No	**NS**	-30.96 to 34.01
Y. (-) vs. E. (+) High	18.69	1.61	No	**NS**	-15.89 to 53.28
Y. (+) Low vs. Y. (+) High	-13.87	1.573	No	**NS**	-40.13 to 12.40
Y. (+) Low vs. E. (-)	-18.51	1.964	No	**NS**	-46.58 to 9.559
Y. (+) Low vs. E. (+) Low	-14.34	1.362	No	**NS**	-45.73 to 17.04
Y. (+) Low vs. E. (+) High	2.822	0.2506	No	**NS**	-30.73 to 36.37
Y. (+) High vs. E. (-)	-4.645	0.4574	No	**NS**	-34.89 to 25.60
Y. (+) High vs. E. (+) Low	-0.4785	0.04274	No	**NS**	-33.82 to 32.87
Y. (+) High vs. E. (+) High	16.69	1.405	No	**NS**	-18.71 to 52.08
E. (-) vs. E. (+) Low	4.167	0.3568	No	**NS**	-30.62 to 38.95
E. (-) vs. E. (+) High	21.33	1.729	No	**NS**	-15.42 to 58.09
E. (+) High vs. E. (+) High	17.17	1.3	No	**NS**	-22.17 to 56.51

*p < 0.05, **p < 0.01, *** p <0.001, **NS**: Not Significant.

Table S4. Associations of age, sex, and HBV DNA status with serum TSH concentrations: results from multiple linear regression and ANCOVA models.

**Multiple Linear Regression**		
**Model**	**R**	**R^2**
1	0.649	0.421

Model Coefficients (TSH)

**Predictor**	**Estimate**	**SE**	**t**	**p**
Intercept	0.8041	0.156	5.141	<.001
Sex: M – F	-0.0912	0.131	-0.697	0.487
HBV DNA group: Low – High	-0.1394	0.132	-1.056	0.293
Age: Younger – Elder	1.2757	0.142	8.984	<.001

ANCOVA

**TSH**	**Sum of Squares**	**df**	**Mean Square**	**F**	**p**
**Sex**	0.34323	1	0.34323	0.66439	0.417
**HBV DNA group**	0.3385	1	0.3385	0.65522	0.42
**Age**	39.59787	1	39.59787	76.64902	<.001
**Sex ∗ HBV DNA group**	0.15542	1	0.15542	0.30085	0.584
**Sex ∗ Age**	0.02682	1	0.02682	0.05191	0.82

**HBV DNA group ∗ Age**	0.0788	1	0.0788	0.15254	0.697
**Sex ∗ HBV DNA group ∗ Age**	0.00116	1	0.00116	0.00225	0.962
**Residuals**	56.82742	110	0.51661		

SE, standard error; ANCOVA, analysis of covariance; HBV, hepatitis B virus.

Table S5. Associations of age, sex, and HBV DNA status with serum FT4 concentrations: results from multiple linear regression and ANCOVA models.

**Multiple Linear Regression**		
**Model**	**R**	**R^2**
1	0.208	0.0431

Model Coefficients (FT4)

Predictor	Estimate	SE	t	p
Intercept	1.0872	0.0567	19.1621	<.001
Sex: M – F	0.0515	0.0475	1.0832	0.281
HBV DNA group: Low – High	7.20E-04	0.048	0.015	0.988
Age: Younger – Elder	0.0486	0.0515	0.943	0.348

ANCOVA

FT4	Sum of Squares	df	Mean Square	F	p
**Sex**	0.04749	1	0.04749	0.7028	0.404
**HBV DNA group**	0.01401	1	0.01401	0.2073	0.65
**Age**	0.07454	1	0.07454	1.103	0.296
**Sex ∗ HBV DNA group**	7.73E-04	1	7.73E-04	0.0114	0.915
**Sex ∗ Age**	0.00958	1	0.00958	0.1417	0.707
**HBV DNA group ∗ Age**	0.07752	1	0.07752	1.1471	0.287
**Sex ∗ HBV DNA group ∗ Age**	0.04397	1	0.04397	0.6506	0.422
**Residuals**	7.43325	110	0.06758		

SE, standard error; ANCOVA, analysis of covariance; HBV, hepatitis B virus.

Table S6. Associations of age, sex, and HBV DNA status with serum CRP concentrations: results from multiple linear regression and ANCOVA models.

**Multiple Linear Regression**		
**Model**	**R**	**R^2**
1	0.208	0.0431

Model Coefficients (CRP)

Predictor	Estimate	SE	t	p
Intercept	0.4957	0.242	2.0488	0.043
Sex: M – F	0.0182	0.203	0.0898	0.929
HBV DNA group: Low – High	0.1865	0.205	0.9103	0.365
Age: Younger – Elder	-0.4613	0.22	-2.0991	0.038

ANCOVA

CRP	Sum of Squares	df	Mean Square	F	p
**Sex**	0.0586	1	0.0586	0.0482	0.827
**HBV DNA group**	2.6778	1	2.6778	2.2009	0.141
**Age**	4.2722	1	4.2722	3.5113	0.064
**Sex ✻ HBV DNA group**	0.0595	1	0.0595	0.0489	0.825
**Sex ✻ Age**	0.3936	1	0.3936	0.3235	0.571
**HBV DNA group ✻ Age**	3.2743	1	3.2743	2.6911	0.104
**Sex ✻ HBV DNA group ✻ Age**	0.1321	1	0.1321	0.1086	0.742
**Residuals**	133.8388	110	1.2167		

SE, standard error; ANCOVA, analysis of covariance; HBV, hepatitis B virus.

Table S7. Associations of age, sex, and HBV DNA status with serum HDL Cholesterol concentrations: results from multiple linear regression and ANCOVA models.

**Multiple Linear Regression**		
**Model**	**R**	**R^2**
1	0.3	0.0903

Model Coefficients (HDL Cholesterol)

Predictor	Estimate	SE	t	p
Intercept	38.8	2.85	13.598	<.001
Sex: M – F	-5.76	2.39	-2.409	0.018
HBV DNA group: Low – High	1.77	2.42	0.733	0.465
Age: Younger – Elder	5.65	2.59	2.181	0.031

ANCOVA

HDL Cholesterol	Sum of Squares	df	Mean Square	F	p
**Sex**	420.7	1	420.7	2.7903	0.098
**HBV DNA group**	39.6	1	39.6	0.2624	0.609
**Age**	712	1	712	4.7224	0.032
**Sex ✻ HBV DNA group**	641.9	1	641.9	4.2575	0.041
**Sex ✻ Age**	761.9	1	761.9	5.0531	0.027
**HBV DNA group ✻ Age**	1015.5	1	1015.5	6.7357	0.011
**Sex ✻ HBV DNA group ✻ Age**	11.7	1	11.7	0.0773	0.782
**Residuals**	16584.7	110	150.8		

SE, standard error; ANCOVA, analysis of covariance; HBV, hepatitis B virus.

Table S8. Associations of age, sex, and HBV DNA status with serum LDL Cholesterol concentrations: results from multiple linear regression and ANCOVA models.

**Multiple Linear Regression**		
**Model**	**R**	**R^2**
1	0.355	0.126

Model Coefficients (LDL Cholesterol)

Predictor	Estimate	SE	t	p
Intercept	69.23595	6.17	11.23	<.001
Sex: M – F	0.00369	5.17	7.14E-04	0.999

HBV DNA group: Low – High	-7.32822	5.22	-1.4	0.163
Age: Younger – Elder	21.50302	5.6	3.84	<.001

ANCOVA					
**LDL Cholesterol**	**Sum of Squares**	**df**	**Mean Square**	**F**	**p**
**Sex**	137.8	1	137.8	0.1773	0.675
**HBV DNA group**	276.6	1	276.6	0.3558	0.552
**Age**	13010.3	1	13010.3	16.7361	<.001
**Sex ✻ HBV DNA group**	800.3	1	800.3	1.0295	0.313
**Sex ✻ Age**	183	1	183	0.2354	0.629
**HBV DNA group ✻ Age**	2907	1	2907	3.7396	0.056
**Sex ✻ HBV DNA group ✻ Age**	15.3	1	15.3	0.0197	0.889
**Residuals**	85511.3	110	777.4		

SE, standard error; ANCOVA, analysis of covariance; HBV, hepatitis B virus.

Table S9. Associations of age, sex, and HBV DNA status with serum Triglyceride concentrations: results from multiple linear regression and ANCOVA models.

Multiple Linear Regression		
**Model**	**R**	**R^2**
1	0.216	0.0467

Model Coefficients (Triglyceride)

Predictor	Estimate	SE	t	p
Intercept	86.936	7.9	11.0015	<.001
Sex: M – F	14.572	6.7	2.1753	0.032
HBV DNA group: Low – High	-4.423	6.79	-0.6516	0.516
Age: Younger – Elder	-0.458	7.3	-0.0628	0.95

ANCOVA					
**Triglyceride**	**Sum of Squares**	**df**	**Mean Square**	**F**	**p**
**Sex**	3521.3	1	3521.3	2.9247	0.09
**HBV DNA group**	53.5	1	53.5	0.0445	0.833
**Age**	41.3	1	41.3	0.0343	0.853
**Sex ✻ HBV DNA group**	22.9	1	22.9	0.019	0.891
**Sex ✻ Age**	694	1	694	0.5765	0.449
**HBV DNA group ✻ Age**	4981	1	4981	4.1372	0.045
**Sex ✻ HBV DNA group ✻ Age**	1041.8	1	1041.8	0.8653	0.354
**Residuals**	121600.2	101	1204		

SE, standard error; ANCOVA, analysis of covariance; HBV, hepatitis B virus.
